# Mechanistic insights into the dimethylsulfoniopropionate synthesis enzyme BurB

**DOI:** 10.1128/aem.01354-25

**Published:** 2025-09-04

**Authors:** Nan Zhang, Yue Lin, Ning Wang, Hai-Yan Cao, Buke Zhang, Ya-Nan Gao, Yu-Zhong Zhang, Chun-Yang Li

**Affiliations:** 1 School of Bioengineering, State Key Laboratory of Biobased Material and Green Papermaking, Qilu University of Technology (Shandong Academy of Sciences) 562796, Jinan, China; 2 MOE Key Laboratory of Evolution and Marine Biodiversity, Frontiers Science Center for Deep Ocean Multispheres and Earth System & College of Marine Life Sciences, Ocean University of China 12591, Qingdao, China; 3 Joint Research Center for Marine Microbial Science and Technology, Shandong University and Ocean University of China, Qingdao, China; 4 Marine Biotechnology Research Center, State Key Laboratory of Microbial Technology, Shandong University 214177 , Qingdao, China; 5 Laboratory for Marine Biology and Biotechnology, Qingdao Collaborative Innovation Center of Marine Science and Technology 554912 , Qingdao, China; Georgia Institute of Technology, Atlanta, Georgia, USA

**Keywords:** DMSP synthesis, *S*-methyltransferase, SET domain, catalytic mechanism, global sulfur cycle

## Abstract

Dimethylsulfoniopropionate (DMSP) is one of the most abundant organosulfur molecules on Earth. It possesses various physiological functions in microorganisms and plays key roles in the global climate regulation. BurB, a SET (Suppressor of variegation, Enhancer of zeste and Trithorax) domain-containing enzyme identified from *Burkholderia thailandensis*, initiates DMSP synthesis by methylating methionine (Met) to *S*-methyl-methionine (SMM), with *S*-adenosyl methionine (SAM) as a methyl donor. Here, the crystal structures of BurB-Met and BurB-SMM-SAM were determined, and the catalytic mechanism of BurB was proposed based on structural and biochemical analyses. BurB is a specific *S*-methyltransferase involved in the DMSP methylation synthesis pathway. The Met molecule is bound in the substrate binding pocket mainly via hydrogen bonding interactions with the main chains of the BurB residues. With the binding of SAM, the loop (Gln37-Ala44) possessing a gating function generates a conformational change and seals the substrate binding pocket, which may promote the subsequent nucleophilic attack of the Met molecule on the methyl group of SAM via the proximity and desolvation mechanism. Our results offer a better understanding of the catalytic mechanisms of SET domain-containing methyltransferases and provide novel insights into DMSP synthesis and the global sulfur cycling.

**IMPORTANCE:**

The organosulfur compound dimethylsulfoniopropionate (DMSP) is an important participant in the global sulfur cycling. DMSP possesses various physiological functions in microorganisms and is also the main precursor of the “climate cooling” gas dimethyl sulfide (DMS). However, studies on the catalytic mechanisms of DMSP synthesis enzymes are limited. BurB, a specific *S*-methyltransferase involved in the DMSP methylation synthesis pathway, catalyzes the conversion of methionine (Met) to *S*-methyl-methionine (SMM), with *S*-adenosyl methionine (SAM) as a methyl donor. Moreover, BurB also represents a new member of the SET (Suppressor of variegation, Enhancer of zeste and Trithorax) domain proteins. Here, we determined the crystal structures of BurB-Met and BurB-SMM-SAM complexes and proposed the catalytic mechanism of BurB based on structural and biochemical analyses. The results offer a better understanding of the catalytic mechanisms of SET domain-containing methyltransferases and provide novel insights into DMSP synthesis.

## INTRODUCTION

The sulfur-containing zwitterion dimethylsulfoniopropionate (DMSP) is one of the most abundant organosulfur molecules on Earth. Approximately 8 billion tons of DMSP are produced annually by marine algae, bacteria, corals, and higher plants (1–4). DMSP possesses various physiological functions (5), such as osmoprotection (6), hydrostatic pressure protection (7), antioxidation (8), and signaling (9). Diverse marine bacteria can concentrate DMSP to high millimolar intracellular levels and catabolize DMSP to produce the climate-active gases dimethyl sulfide (DMS) and methanethiol (MeSH) (1, 10–13). In the air, the oxidation products of DMS and, probably, MeSH can participate in the formation of cloud condensation nuclei, which may further influence global weather and climate (14–16). Moreover, DMSP is also a potential precursor of the greenhouse gas methane (17).

**Fig 1:**
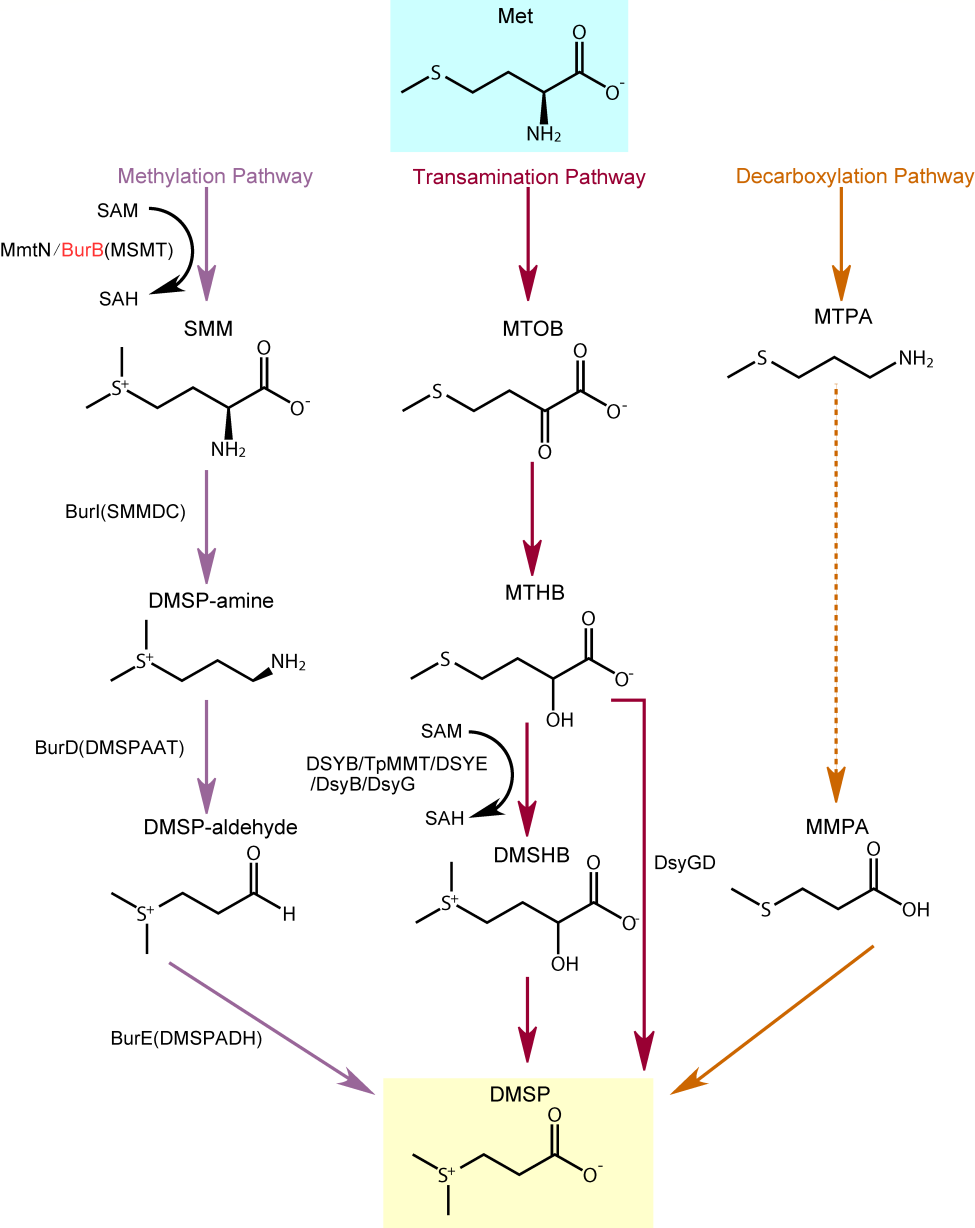
Three DMSP biosynthetic pathways. The DMSP synthesis enzyme BurB focused in this study is colored in red. Dashed lines indicate the unconfirmed steps in the decarboxylation pathway. Met, methionine; SMM, *S*-methyl-methionine; SAH, *S*-adenosyl homocysteine; SAM, *S*-adenosyl methionine; MTOB, 4-methylthio-2-oxobutyrate; MTHB, 4-methylthio-2-hydroxybutyrate; DMSHB, 4-dimethylsulfonio-2-hydroxybutyrate; MTPA, 3-methylthiopropylamine; MMPA, methylmercaptopropionate.

Three DMSP synthesis pathways have been reported with the amino acid methionine (Met) as a precursor (Fig. 1): the methylation pathway in bacteria and angiosperms (18–21); the transamination pathway utilized by diverse marine algae, bacteria, and corals (22–24); and the decarboxylation pathway identified in one dinoflagellate (25). In the methylation pathway, Met is first methylated to produce *S*-methyl-methionine (SMM), which is subsequently metabolized to DMSP-amine, DMSP-aldehyde, and finally DMSP (18) (Fig. 1). Three enzymes catalyzing the conversion of Met to SMM have been identified, including MmtN from saltmarsh sediment bacteria (19), BurB from *Burkholderia thailandensis* (21), and MSMT from *Streptomyces mobaraensis* (20). The enzymes catalyzing the downstream metabolism of SMM have also been identified, naming BurI, BurD, and BurE in *B. thailandensis* and SMMDC, DMSPAAT, and DMSPADH in *S. mobaraensis* (20, 21) (Fig. 1). In fact, the DMSP synthesis in *B. thailandensis* and *S. mobaraensis* is similar, with BurB, BurI, BurD, and BurE being homologous enzymes of MSMT, SMMDC, DMSPAAT, and DMSPADH, respectively.

In the transamination DMSP synthesis pathway, Met is first deaminated to 4-methylthio-2-oxobutyrate (MTOB), which is subsequently metabolized to 4-methylthio-2-hydroxybutyrate (MTHB), 4-dimethylsulfonio-2-hydroxybutyrate (DMSHB), and finally DMSP (23) (Fig. 1). Six enzymes in this pathway have been identified. They are isozymes catalyzing the methylation of MTHB to DMSHB, among which DsyGD also exhibits DMSHB decarboxylation activity (3, 22, 23, 26) (Fig. 1). The first step of the decarboxylation pathway is the decarboxylation of DMSP to produce 3-methylthiopropylamine (MTPA) (25). The subsequent steps in this pathway have not been confirmed, and no enzymes have been identified yet (Fig. 1).

Among the DMSP synthesis enzymes, the catalytic mechanisms of DsyB and MmtN have been proposed based on structural and biochemical analyses (27, 28). DsyB forms a homodimer in solution and catalyzes the conversion of MTHB to DMSHB using a proximity and desolvation mechanism (PD mechanism) (28). DSYB, the eukaryotic homolog of DsyB, likely adopts a similar catalytic mechanism to DsyB (28). MmtN functions as a homotrimer in solution and also adopts the PD mechanism to catalyze the methylation of Met to SMM (27).

The pathogen *B. thailandensis* produces DMSP as a precursor of malleicyprol virulence factors (21, 29). Malleicyprols may contribute to evading eukaryotic predators or killing microbial competitors in the environment (30). In fact, acting as a building block in bacterial secondary metabolism is a novel function of DMSP (21). Physiological analysis indicated that the gene *burB* is essential for *B. thailandensis* to produce DMSP (21). BurB is a *S*-adenosyl methionine (SAM)-dependent Met *S*-methyltransferase containing a SET (Suppressor of variegation, Enhancer of zeste and Trithorax) domain, and belongs to the class V of the methyltransferase superfamilies (21, 31, 32). The SET domain proteins are well known for their roles in methylating histone lysines in eukaryotes (32–35), and BurB represents a new member of the SET domain proteins. Although BurB and MmtN catalyze the same reaction in the methylation DMSP synthesis pathway, no significant similarity was found between their amino acid sequences by BLASTP analysis (36). The catalytic mechanism of BurB converting Met to SMM is still unknown due to the absence of structural data.

In this study, the *burB* gene originating from *B. thailandensis* was overexpressed in *Escherichia coli*, and the recombinant BurB proteins were purified and characterized. The crystal structures of BurB-Met and BurB-SMM-SAM complexes were determined by using X-ray crystallography. Based on structural and mutation analysis, the catalytic mechanism of BurB is proposed.

## RESULTS AND DISCUSSION

### Expression and characterization of BurB from *B. thailandensis*

**Fig 2:**
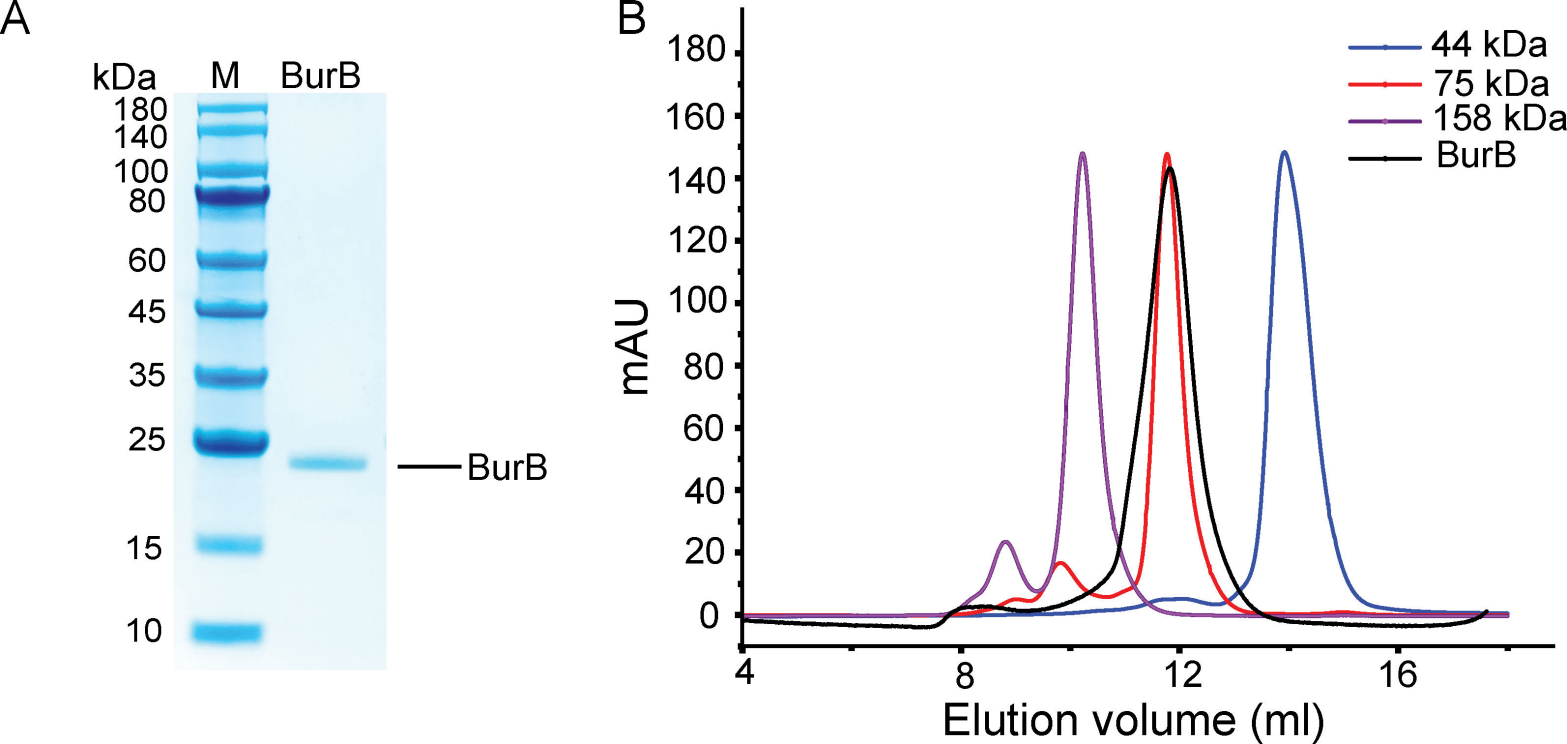
Expression and purification of the recombinant BurB from *B. thailandensis*. (**A**) SDS-PAGE analysis of the purified BurB protein. (**B**) Gel filtration analysis of BurB. Ovalbumin (44,000 Da; Cytiva), conalbumin (75,000 Da; Cytiva) and aldolase (158,000 Da; Cytiva) were used as markers. The predicted molecular mass of BurB is 19.05 kDa (http://web.expasy.org/compute_pi/).

The *burB* gene from *B. thailandensis* contains 519 nucleotides and codes a protein of 172 amino acids. The full-length *burB* gene was synthesized, overexpressed in *E. coli* BL21 (DE3) cells, and the recombinant BurB proteins were purified (Fig. 2A). The predicted molecular mass of BurB is 19.05 kDa (http://web.expasy.org/compute_pi/), and gel filtration analysis indicated that BurB maintains a tetramer in solution (Fig. 2B).

**Fig 3:**
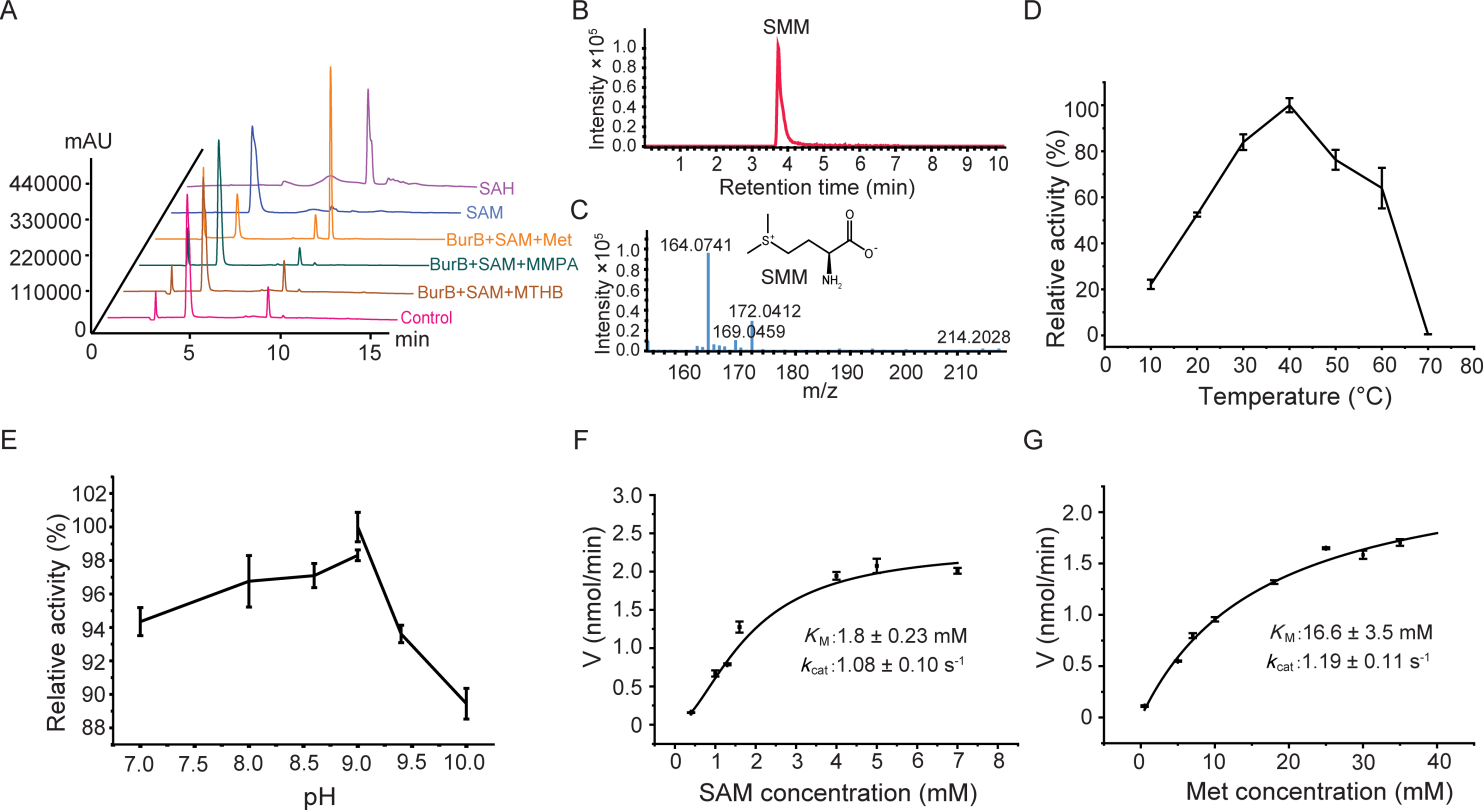
Characterization of the recombinant BurB. (**A**) The substrate specificity of BurB. The methylation activities of BurB towards Met, MMPA, and MTHB were detected by HPLC at 260 nm. The reaction system without BurB was used as the control. (**B**) A total ion chromatogram of SMM (extracted *m/z* = 164) produced by BurB *S*-methylation of Met via LC–MS. (**C**) The mass spectra of SMM produced by BurB *S*-methylation of Met via LC–MS. (**D**) Effect of temperature on the enzymatic activity of BurB. The activity of BurB at 40°C (0.36 µmol/min/mg) was defined as 100%. (**E**) Effect of pH on the enzymatic activity of BurB. The activity of BurB at pH 9.0 (0.36 μmol/min/mg) was defined as 100%. (**F**) A non-linear fit curve for SAM demethylation by BurB. (**G**) A non-linear fit curve for Met methylation by BurB. The error bar represents standard deviation of triplicate experiments.

The enzymatic activity of the recombinant BurB was verified by high-performance liquid chromatography (HPLC) and liquid chromatography-mass spectrometry (LC-MS) assays. With SAM as the methyl donor, the purified BurB catalyzed the *S*-methylation of Met and produced *S*-adenosyl homocysteine (SAH) (Fig. 3A) and SMM (Fig. 3B and C). The optimal temperature of BurB enzymatic activity was 40°C (Fig. 3D), and the optimal pH was 9.0 (Fig. 3E). It is notable that the strain *B. thailandensis* was originally isolated from soil samples in Thailand (37), and the optimal temperature and pH are relatively high compared with the natural environment where *B. thailandensis* was originally isolated. However, BurB maintained over 80% of its highest enzymatic activity at 30°C (Fig. 3D) and approximately 95% of its highest enzymatic activity at pH 7.0–8.0 (Fig. 3E), indicating that BurB should function properly in the environments. *B. thailandensis* BurB exhibited a *K*_M_ value of 1.8 mM towards SAM (Fig. 3F) and 16.6 mM towards Met (Fig. 3G) at pH 9.0 and 40°C. MmtN, an isoenzyme of BurB, is also reported to possess high millimolar *K*_M_ values towards SAM and Met (19, 27). Substrate specificity analysis revealed that BurB was only active towards Met but not MTHB, an intermediate in the DMSP transamination synthesis pathway (23), or methylmercaptopropionate (MMPA), a possible intermediate in the DMSP decarboxylation synthesis pathway (25) (Fig. 3A). These results suggest that BurB is a specific *S*-methyltransferase involved in the DMSP methylation synthesis pathway (Fig. 1).

### Overall structure of BurB

**Fig 4:**
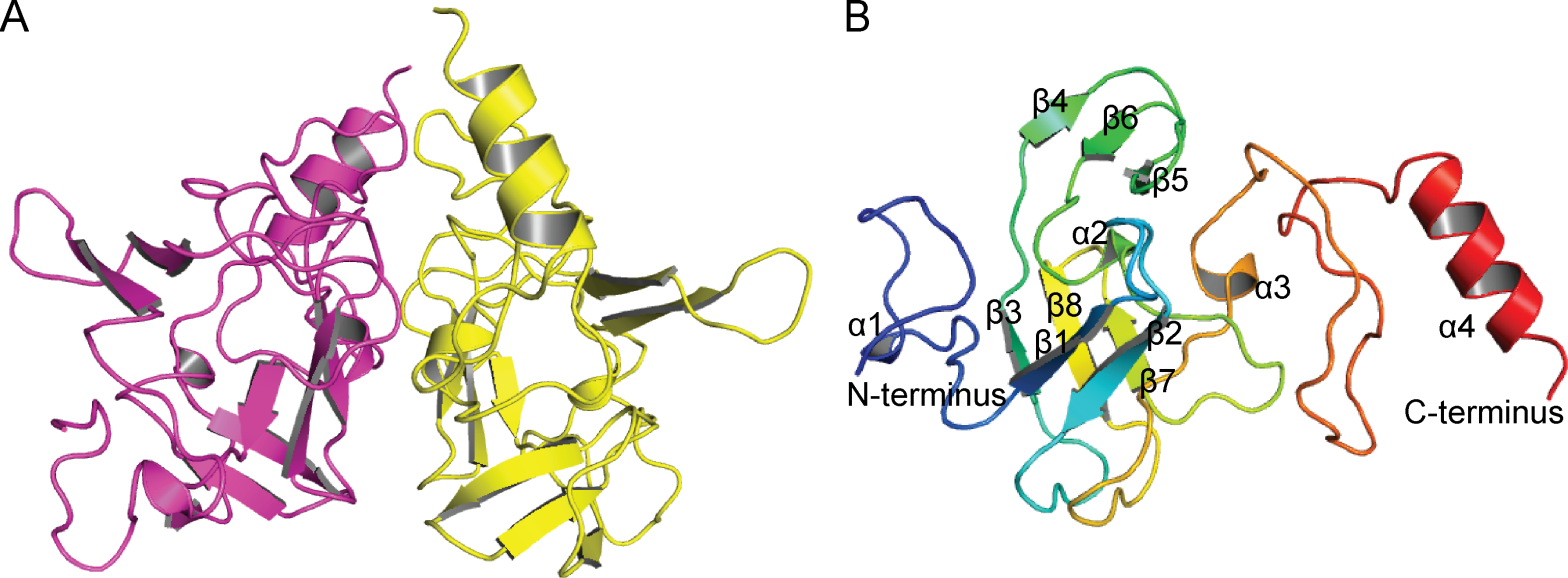
Overall structure analysis of BurB. (**A**) Overall structure of the BurB-Met complex. There are two BurB molecules in an asymmetric unit, which are colored in magenta and yellow, respectively. (**B**) The structure of one BurB monomer, which is composed of eight β-sheets and four α-helices.

To probe the catalytic mechanism of BurB, we solved the crystal structures of the BurB-Met and BurB-SMM-SAM complexes (Table 1). The crystals of the BurB-Met complex belong to the *C*121 space group, with two BurB molecules arranged as a dimer in an asymmetric unit (Fig. 4A). Each BurB monomer consists of eight β-sheets and four α-helices (Fig. 4B). The crystals of BurB-SMM-SAM complex belong to the *I*222 space group, and there is only one BurB molecule in an asymmetric unit. The overall structures of BurB-Met and BurB-SMM-SAM complexes are similar, with a root mean square deviation (RMSD) of 0.33 Å between them.

**TABLE 1:** Crystallographic data collection and refinement

Parameters	BurB-Met complex	BurB-SMM-SAM complex
Diffraction data		
Space group	*C*121	*I*222
Unit cell		
a, b, c (Å)	123.0, 67.4, 59.3	63.1, 67.2, 103.5
α, β, γ (°)	90.0, 117.5, 90.0	90.0, 90.0, 90.0
Resolution range (Å)	50.00–2.15 (2.19–2.15)^*a*^	50.00–2.30 (2.34–2.30)
Redundancy	6.2 (5.3)	12.9 (12.7)
Completeness (%)	99.3 (96.9)	98.7 (98.4)
*R*_merge_^*b*^	0.1 (0.4)	0.1 (0.4)
*I*/σ*I*	27.0 (3.6)	30.0 (4.8)
Refinement statistics		
R-factor	0.21	0.19
Free R-factor	0.25	0.22
RMSD from ideal geometry		
Bond lengths (Å)	0.008	0.013
Bond angles (°)	1.10	1.36
Ramachandran plot (%)		
Favored	94.9	97.5
Allowed	5.1	2.5
Outliers	0.0	0.0
Overall B-factors (Å^2^)	57.7	43.0

^*a*^ Numbers in parentheses refer to data in the highest resolution shell.

^*b*^ *R*_merge_=∑*_hkl_*∑*_i_*|*I*(*hkl*)*_i_* - < *I*(*hkl*)>|/∑*_hkl_*∑*_i_I*(*hkl*)*_i_*, where *I* is the observed intensity, <*I*(*hkl*)> represents the average intensity, *I*(*hkl*)*_i_* represents the observed intensity of each unique reflection.

### The substrate binding sites of BurB

**Fig 5:**
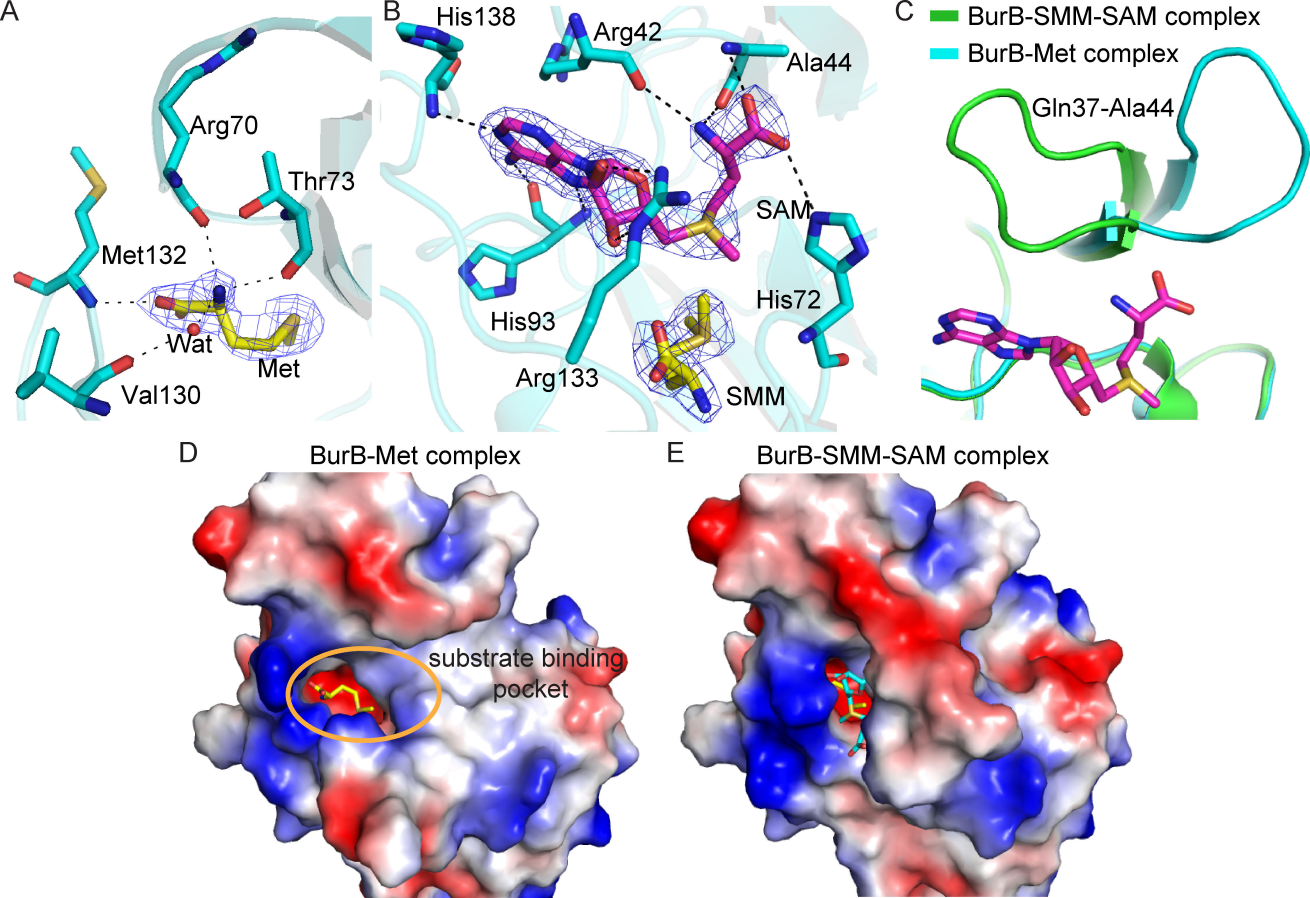
The substrate binding sites of BurB. (**A**) BurB residues involved in Met binding. The residues are shown as cyan sticks, and the Met molecule as yellow sticks. The 2*F*_o_-*F*_c_ densities for Met are contoured in blue at 2.0 σ. (**B**) BurB residues involved in SAM binding. The residues are shown as cyan sticks, the SAM molecule as purple sticks, and the SMM molecule as yellow sticks. The 2*F*_o_-*F*_c_ densities for SAM and SMM are contoured in blue at 2.0 σ. (**C**) Gating function analysis of the loop Gln37-Ala44. The BurB-SMM-SAM complex is colored in green, and the BurB-Met complex in cyan. The SAM molecule is shown as purple sticks. (**D**) Electrostatic surface of the BurB-Met complex. The Met molecule is shown as yellow sticks. The substrate binding pocket is marked as a yellow circle. (**E**) Electrostatic surface of the BurB-SMM-SAM complex. The SMM molecule is shown as yellow sticks, and SAM as cyan sticks.

The electron density map of the BurB-Met complex suggested that there is a Met molecule bound in the substrate binding site of BurB mainly by hydrogen bond interactions (Fig. 5A). Residues Arg70 and Thr73 can form hydrogen bonds with the nitrogen atom of Met. Met132 interacts with the carboxyl group of Met. Moreover, Val130 participates in Met binding by hydrogen bonds via a water molecule (Fig. 5A). It is noteworthy that all the residues interact with Met via their main chains, likely to enable a relatively stable Met’s binding pattern irrespective of potential mutations.

In the BurB-SMM-SAM complex, the SMM molecule is located in the same position as Met in the BurB-Met complex. In addition to the SMM molecule, the SAM molecule was also clearly observed in the BurB-SMM-SAM complex structure (Fig. 5B). Residues His93 and His138 form hydrogen bonds with the SAM adenine ring. The side chain of Arg133 interacts with the ribose ring. The amino and carboxyl groups of SAM contact with residues Arg42, Ala44, and His72 via hydrogen bonds (Fig. 5B).

By comparing the structures of BurB-Met and BurB-SMM-SAM complexes, we found that a loop (Gln37-Ala44) possesses a gating function for SAM entry (Fig. 5C). In the BurB-Met complex, the Met molecule is accessible to the surface (Fig. 5D), suggesting that BurB possesses an open conformation. After the SAM molecule enters into the substrate-binding pocket, the loop (Gln37-Ala44) generates a conformational change (Fig. 5C through E) and participates in binding SAM via the residues Arg42 and Ala44 (Fig. 5B). This conformational change seals the substrate binding pocket (Fig. 5E), which may promote the subsequent methylation of Met.

Conformational changes during catalysis have been reported in DMSP synthesis enzymes MmtN and DsyB (27, 28). Both MmtN and DsyB are methyltransferases and depend on SAM as a methyl donor. The binding of the SAM molecule is the first step during the catalysis of these two enzymes, which triggers their conformational change to form the binding pocket for Met (for MmtN) or MTHB (for DsyB) (27, 28). However, the Met molecule is located deeper in the substrate binding pocket of BurB (Fig. 5D and E), and the binding of SAM seals the substrate binding pocket. Thus, the binding of the Met molecule may occur before the binding of SAM in BurB, which is different from MmtN and DsyB. In fact, the binding pattern of substrates in BurB is similar to other reported SET domain-containing methyltransferases (31, 35, 38). It was proposed that this binding pattern would permit multiple rounds of lysine methylation without releasing the histone substrate from the SET domain (38–40).

### Mechanism of Met *S***-**methylation catalyzed by BurB

**Fig 6:**
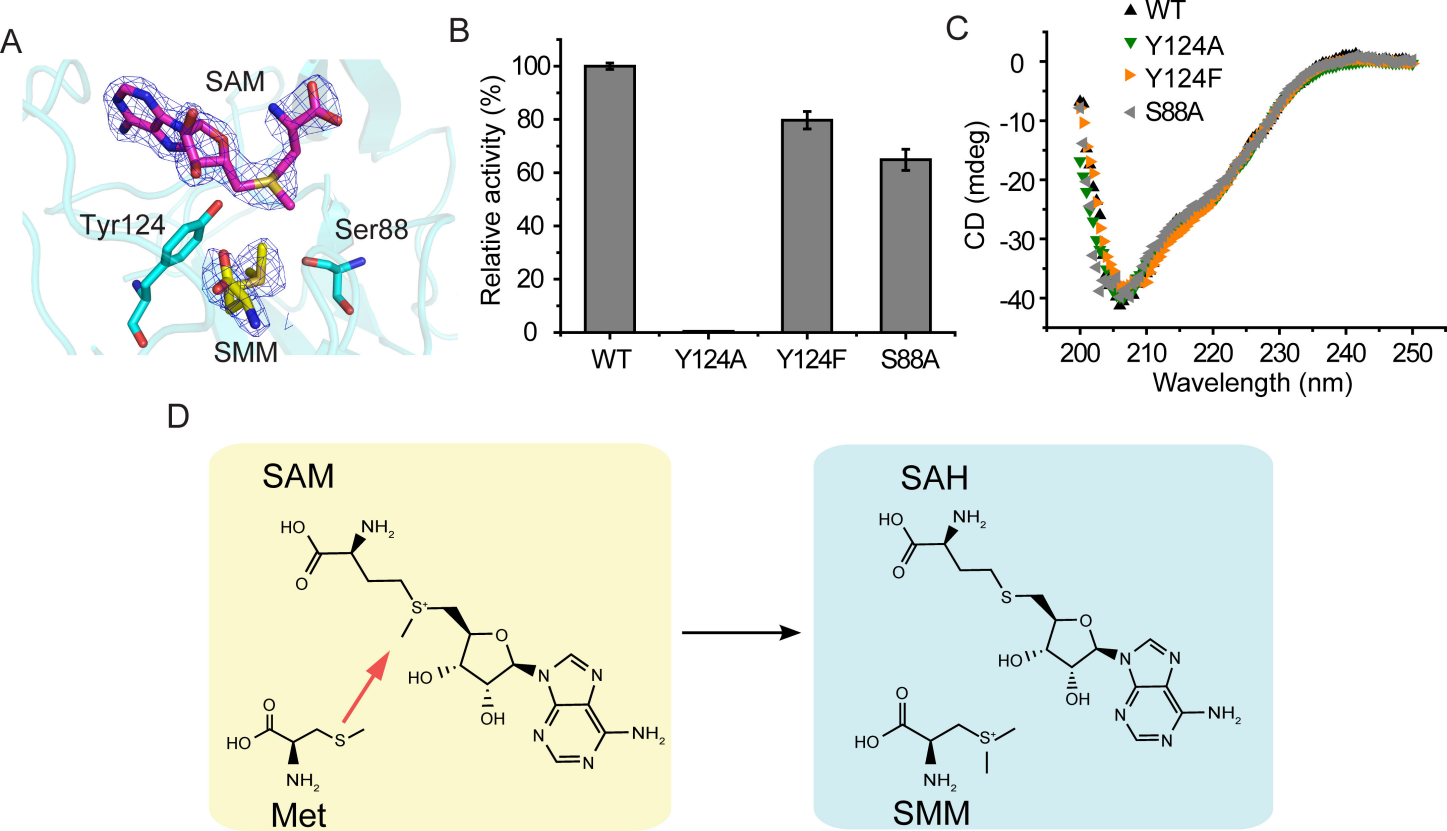
The catalytic mechanism of BurB. (**A**) Structural analysis of the catalytic residues in BurB. The BurB residues Tyr124 and Ser88 are shown as cyan sticks, SAM in purple sticks, and SMM in yellow sticks. The 2*F*_o_-*F*_c_ densities for SAM and SMM are contoured in blue at 2.0 σ. (**B**) Enzymatic activities of WT BurB and its mutants. The activity of WT BurB was defined as 100%. The error bar represents standard deviation of triplicate experiments. (**C**) Circular dichroism spectra of WT BurB and its mutants. (**D**) The proposed catalytic mechanism of BurB.

The Met *S*-methylation to produce SMM catalyzed by BurB is a typical S_N_2 nucleophilic substitution (41, 42). Structural analysis of the BurB-SMM-SAM complex indicated that either residue Tyr124 or Ser88, which are located between the SMM molecule and the SAM molecule (Fig. 6A), may act as the catalytic residue. To probe their function, we mutated Tyr124 to alanine and phenylalanine, and Ser88 to alanine. Then, the enzyme activities of the mutants were measured. The results showed that compared with the wild-type (WT) BurB, the enzyme activity of Y124A almost completely disappeared, while the Y124F and S88A enzymes still retained over 60% of their activity (Fig. 6B), indicating that the residues Tyr124 and Ser88 are not the catalytic residues of BurB. The loss of Y124A enzyme activity was likely due to the microenvironmental change of the active center caused by the replacement of the aromatic side chain. Circular dichroism (CD) spectral analysis suggested that the secondary structures of the mutants were similar to that of WT BurB (Fig. 6C), indicating that the decreases in the BurB enzymatic activities were caused by amino acid replacement rather than structural changes.

Three distinct mechanisms for SAM-dependent methyltransferases have been reported: the PD mechanism, general acid/base-mediated mechanism, and metal-dependent mechanism (43). Structural and biochemical analyses suggested that BurB possesses the PD mechanism for catalysis, which is also adopted by MmtN and DsyB (27, 28). During catalysis, Met is firstly bound in the substrate binding pocket, while BurB maintains an open conformation for the subsequent binding of SAM (Fig. 5A and D). After SAM enters the substrate binding pocket, BurB generates a conformational change to form the closed conformation (Fig. 5C and E), which brings the sulfur atom of Met in close proximity to the SAM methyl group and allows the nucleophilic attack on the methyl group (Fig. 6D). The generated SMM and SAH are released after the reaction, and BurB can rebind the substrates from the intracellular environment in preparation for the next reaction.

### Conclusion

DMSP contributes significantly to the global sulfur cycle. In this study, we solved the crystal structure of BurB, a SET domain-containing enzyme involved in DMSP synthesis, and proposed its catalytic mechanism based on structural and biochemical analyses. The results provide novel insights into DMSP synthesis and lead to a better understanding of the catalytic mechanisms of SET domain-containing methyltransferases.

## MATERIALS AND METHODS

### Gene cloning, synthesis, and point mutation

The full-length *burB* gene from *B. thailandensis* was synthesized by the Beijing Genomics Institute (China) and then subcloned into the pET-22b (Novagen, America) vector with a C-terminal His-tag. Site-directed mutations in BurB were introduced by the PCR-based method with the QuikChange mutagenesis kit II (Agilent, America) and were verified by DNA sequencing. Primers used in this study are listed in Table 2.

**TABLE 2:** Primers used in this study

Primer	Sequence (5′−3′)
Y124A-F	CGGTGCTCGCAGCATCCATGGTCAGCATTTCGCC
Y124A-R	GGCGAAATGCTGACCATGGATGCTGCGAGCACCG
Y124F-F	CGGTGCTCGCAAAATCCATGGTCAGCATTTCGC
Y124F-R	GCGAAATGCTGACCATGGATTTTGCGAGCACCG
S88A-F	AGCAGCAGGCCGGCAAAATGCGGATCATACAGATGGG
S88A-R	CCCATCTGTATGATCCGCATTTTGCCGGCCTGCTGCT

### Protein expression and purification

The BurB proteins and its mutants were overexpressed in *E. coli* BL21(DE3) cells, which were cultured in lysogeny broth (LB) medium containing 100 µg/mL ampicillin at 37°C. When the optical density of the culture at 600 nm (OD_600_) reached 0.6–0.8, 0.4 mM isopropyl β-*D*-1-thiogalactopyranoside (IPTG) was added, and the cells were then incubated at 16°C for 14–16 h. Bacteria cells were collected by centrifugation (6,000 rpm, 15 min, 4°C), resuspended with lysis buffer (100 mM NaCl, 0.5% glycerol, 50 mM Tris-HCl, pH 8.0), and then fractured by the high-pressure crusher machine (JNBIO, China). The proteins were first purified by affinity chromatography on a Ni^2+^-NTA column (Cytiva, America), and then fractionated by gel filtration on a Superdex G200 column (Cytiva, America) with the buffer containing 100 mM NaCl and 10 mM Tris-HCl (pH 8.0).

### Enzyme activity assay and product identification

The enzymatic activity of BurB was measured by using high-performance liquid chromatography (HPLC) (Shimadzu, Japan) to detect the production of SAH at 260 nm on a SunFire C18 column (Waters, Ireland), as previously described (27). The SAM standard was purchased from New England Biolabs (America), SAH, MTHB, and Met were from Sigma-Aldrich (America), and MMPA was from Macklin Biochemical Co., Ltd. (China). BurB (at a final concentration of 0.15 µM), SAM (at a final concentration of 0.6 mM) and Met, MTHB, or MMPA (at a final concentration of 10 mM), were mixed with the reaction buffer containing 200 mM Tris-HCl (pH 8.0) in a total volume of 100 µL. After the mixtures were incubated at 40°C for 30 min, 10% (v/v) HClO_4_ was added to stop the reaction. The enzymatic activity of BurB was measured within the linear range with reaction time and enzyme concentration. The reaction mixture without BurB proteins was set up as the control. To determine the optimal temperature for BurB enzymatic activity, reaction mixtures were incubated at 10°C–70°C (with a 10°C interval) for 30 min. The optimal pH was measured at 40°C using 200 mM Tris-HCl buffer for pH 7.0–9.0 and 200 mM Glycine-NaOH buffer for pH 9.0–10.0. The kinetic parameters of BurB were measured at pH 9.0 and 40°C.

### Liquid chromatography–mass spectrometry (LC–MS) analysis

LC–MS was performed by using a Dionex HPLC system, as previously described (27). A Phenomenex Luna NH2 column (100 × 2 mm) was used to analyze the samples in the hydrophilic interaction chromatography mode. The conditions of MS spray chamber were capillary voltage 4.5 kV, oven temperature 30°C, desolvation temperature 180°C and nebulizing gas flow 1.0 L/min. The targeted mass transition corresponded to the [M + H]^+^ of SMM (*m/z* = 164) in positive mode.

### Spectral analysis of circular dichroism (CD)

CD spectra for BurB and its mutants were analyzed in a 0.1 cm-path length cell on a JASCO J-1500 Spectrometer (Japan). The final concentrations of proteins were adjusted to 20 µM with 10 mM Tris-HCl (pH 8.0) and 100 mM NaCl. The spectra were recorded from 250 to 200 nm at a scan speed of 200 nm/min.

### Crystallization and data collection

The purified BurB proteins were concentrated to ~7.5 mg/mL in the buffer containing 10 mM Tris-HCl and 100 mM NaCl. To obtain crystals of BurB-Met complex and BurB-SMM-SAM complex, the BurB proteins were incubated with Met (10 mM) or SMM (10 mM) and SAM (1 mM) for 30 min, respectively. The initial crystallizations of the proteins were carried out at 18°C using the sitting-drop vapor diffusion method. Crystals of BurB-Met were obtained in hanging drops containing 0.2 M ammonium sulfate, 0.1 M Tris (pH 8.5), and 15% (w/v) polyethylene glycol (PEG) 8000 after 1-week incubation. Crystals of BurB-SMM-SAM were obtained in hanging drops containing 0.2 M ammonium sulfate, 0.1 M Tris (pH 8.4), and 11% (w/v) PEG 8000. We have also tried different crystallization conditions for BurB-SAM complex, BurB-Met-SAH complex, and BurB-SMM-SAH complex, but none of them yielded diffraction-quality crystals. X-ray diffraction data were collected on the BL17U1&BL18U1 beamlines at Shanghai Synchrotron Radiation Facility. The initial diffraction data were processed using the HKL3000 program with default settings (44).

### Structure determination and refinement

The structures of BurB-Met and BurB-SMM-SAM complexes were determined by molecular replacement with the CCP4 program (45) using a structure predicted by AlphaFold2 (46) as a search model. Structure refinement was conducted using *Phenix* (47), and real space refinement was performed using Coot (48). Images of protein structures were created using PyMOL (https://www.pymol.org/).

## Data Availability

The structures of BurB-Met complex and BurB-SMM-SAM complex have been deposited in the Protein Data Bank under the accession codes 9L4T and 9L4V, respectively.

## References

[1] Hopkins FE, Archer SD, Bell TG, Suntharalingam P, Todd JD. 2023. The biogeochemistry of marine dimethylsulfide. Nat Rev Earth Environ 4:361–376. doi:10.1038/s43017-023-00428-7

[2] Galí M, Devred E, Levasseur M, Royer S-J, Babin M. 2015. A remote sensing algorithm for planktonic dimethylsulfoniopropionate (DMSP) and an analysis of global patterns. Remote Sens Environ 171:171–184. doi:10.1016/j.rse.2015.10.012

[3] Wang J, Curson ARJ, Zhou S, Carrión O, Liu J, Vieira AR, Walsham KS, Monaco S, Li C-Y, Dong Q-Y, Wang Y, Rivera PPL, Wang X-D, Zhang M, Hanwell L, Wallace M, Zhu X-Y, Leão PN, Lea-Smith DJ, Zhang Y-Z, Zhang X-H, Todd JD. 2024. Alternative dimethylsulfoniopropionate biosynthesis enzymes in diverse and abundant microorganisms. Nat Microbiol 9:1979–1992. doi:10.1038/s41564-024-01715-9 38862603 PMC11306096

[4] Payet RD, Bilham LJ, Kabir SMT, Monaco S, Norcott AR, Allen MGE, Zhu X-Y, Davy AJ, Brearley CA, Todd JD, Miller JB. 2024. Elucidation of Spartina dimethylsulfoniopropionate synthesis genes enables engineering of stress tolerant plants. Nat Commun 15:8568. doi:10.1038/s41467-024-51758-z 39384757 PMC11464771

[5] Li C-Y, Cao H-Y, Payet RD, Todd JD, Zhang Y-Z. 2024. Dimethylsulfoniopropionate (DMSP): from biochemistry to global ecological significance. Annu Rev Microbiol 78:513–532. doi:10.1146/annurev-micro-041222-024055 39231449

[6] Kirst GO. 1990. Salinity tolerance of eukaryotic marine algae. Annu Rev Plant Physiol Plant Mol Biol 41:21–53. doi:10.1146/annurev.pp.41.060190.000321

[7] Zheng Y, Wang J, Zhou S, Zhang Y, Liu J, Xue C-X, Williams BT, Zhao X, Zhao L, Zhu X-Y, Sun C, Zhang H-H, Xiao T, Yang G-P, Todd JD, Zhang X-H. 2020. Bacteria are important dimethylsulfoniopropionate producers in marine aphotic and high-pressure environments. Nat Commun 11:4658. doi:10.1038/s41467-020-18434-4 32938931 PMC7494906

[8] Sunda W, Kieber DJ, Kiene RP, Huntsman S. 2002. An antioxidant function for DMSP and DMS in marine algae. Nature 418:317–320. doi:10.1038/nature00851 12124622

[9] Li CY, Cao HY, Curson ARJ, Wang P, Todd JD, Zhang YZ. 2023. Dimethylsulfoniopropionate and its catabolites are important chemical signals mediating marine microbial interactions. Trends Microbiol 31:992–994. doi:10.1016/j.tim.2023.07.004 37481345

[10] Li C-Y, Mausz MA, Murphy A, Zhang N, Chen X-L, Wang S-Y, Gao C, Aguilo-Ferretjans MM, Silvano E, Lidbury I, Fu H-H, Todd JD, Chen Y, Zhang Y-Z. 2023. Ubiquitous occurrence of a dimethylsulfoniopropionate ABC transporter in abundant marine bacteria. ISME J 17:579–587. doi:10.1038/s41396-023-01375-3 36707613 PMC10030565

[11] Sun J, Todd JD, Thrash JC, Qian Y, Qian MC, Temperton B, Guo J, Fowler EK, Aldrich JT, Nicora CD, Lipton MS, Smith RD, De Leenheer P, Payne SH, Johnston AWB, Davie-Martin CL, Halsey KH, Giovannoni SJ. 2016. The abundant marine bacterium Pelagibacter simultaneously catabolizes dimethylsulfoniopropionate to the gases dimethyl sulfide and methanethiol. Nat Microbiol 1:16065. doi:10.1038/nmicrobiol.2016.65 27573103

[12] Reisch CR, Moran MA, Whitman WB. 2008. Dimethylsulfoniopropionate-dependent demethylase (DmdA) from Pelagibacter ubique and Silicibacter pomeroyi. J Bacteriol 190:8018–8024. doi:10.1128/JB.00770-08 18849431 PMC2593244

[13] Li CY, Wang XJ, Chen XL, Sheng Q, Zhang S, Wang P, Quareshy M, Rihtman B, Shao X, Gao C, Li FC, Li SY, Zhang WP, Zhang XH, Yang GP, Todd JD, Chen Y, Zhang YZ. 2021. A novel ATP dependent dimethylsulfoniopropionate lyase in bacteria that releases dimethyl sulfide and acryloyl-CoA. Elife 10:e64045. doi:10.7554/eLife.64045 33970104 PMC8163506

[14] Charlson RJ, Lovelock JE, Andreae MO, Warren SG. 1987. Oceanic phytoplankton, atmospheric sulphur, cloud albedo and climate. Nature 326:655–661. doi:10.1038/326655a0

[15] Vallina SM, Simó R. 2007. Strong relationship between DMS and the solar radiation dose over the global surface ocean. Science 315:506–508. doi:10.1126/science.1133680 17255509

[16] Todd JD, Rogers R, Li YG, Wexler M, Bond PL, Sun L, Curson ARJ, Malin G, Steinke M, Johnston AWB. 2007. Structural and regulatory genes required to make the gas dimethyl sulfide in bacteria. Science 315:666–669. doi:10.1126/science.1135370 17272727

[17] Damm E, Kiene RP, Schwarz J, Falck E, Dieckmann G. 2008. Methane cycling in Arctic shelf water and its relationship with phytoplankton biomass and DMSP. Mar Chem 109:45–59. doi:10.1016/j.marchem.2007.12.003

[18] Hanson AD, Rivoal J, Paquet L, Gage DA. 1994. Biosynthesis of 3-dimethylsulfoniopropionate in Wollastonia biflora (L.) DC. (evidence that S-methylmethionine is an intermediate). Plant Physiol 105:103–110. doi:10.1104/pp.105.1.103 8029347 PMC159334

[19] Williams BT, Cowles K, Bermejo Martínez A, Curson ARJ, Zheng Y, Liu J, Newton-Payne S, Hind AJ, Li C-Y, Rivera PPL, Carrión O, Liu J, Spurgin LG, Brearley CA, Mackenzie BW, Pinchbeck BJ, Peng M, Pratscher J, Zhang X-H, Zhang Y-Z, Murrell JC, Todd JD. 2019. Bacteria are important dimethylsulfoniopropionate producers in coastal sediments. Nat Microbiol 4:1815–1825. doi:10.1038/s41564-019-0527-1 31427729

[20] Liao C, Seebeck FP. 2019. In vitro reconstitution of bacterial DMSP biosynthesis. Angew Chem Int Ed 58:3553–3556. doi:10.1002/anie.201814662 30609124

[21] Trottmann F, Ishida K, Franke J, Stanišić A, Ishida‐Ito M, Kries H, Pohnert G, Hertweck C. 2020. Sulfonium acids loaded onto an unusual thiotemplate assembly line construct the cyclopropanol warhead of a Burkholderia virulence factor. Angew Chem Int Ed 59:13511–13515. doi:10.1002/anie.202003958 PMC7496086 32314848

[22] Curson ARJ, Liu J, Bermejo Martínez A, Green RT, Chan Y, Carrión O, Williams BT, Zhang S-H, Yang G-P, Bulman Page PC, Zhang X-H, Todd JD. 2017. Dimethylsulfoniopropionate biosynthesis in marine bacteria and identification of the key gene in this process. Nat Microbiol 2:17009. doi:10.1038/nmicrobiol.2017.9 28191900

[23] Curson ARJ, Williams BT, Pinchbeck BJ, Sims LP, Martínez AB, Rivera PPL, Kumaresan D, Mercadé E, Spurgin LG, Carrión O, Moxon S, Cattolico RA, Kuzhiumparambil U, Guagliardo P, Clode PL, Raina J-B, Todd JD. 2018. DSYB catalyses the key step of dimethylsulfoniopropionate biosynthesis in many phytoplankton. Nat Microbiol 3:430–439. doi:10.1038/s41564-018-0119-5 29483657

[24] Raina J-B, Tapiolas DM, Forêt S, Lutz A, Abrego D, Ceh J, Seneca FO, Clode PL, Bourne DG, Willis BL, Motti CA. 2013. DMSP biosynthesis by an animal and its role in coral thermal stress response. Nature 502:677–680. doi:10.1038/nature12677 24153189

[25] Kitaguchi H, Uchida A, Ishida Y. 1999. Purification and characterization of L-methionine decarboxylase from Crypthecodinium cohnii. Fish Sci 65:613–647. doi:10.2331/fishsci.65.613

[26] Kageyama H, Tanaka Y, Shibata A, Waditee-Sirisattha R, Takabe T. 2018. Dimethylsulfoniopropionate biosynthesis in a diatom Thalassiosira pseudonana: identification of a gene encoding MTHB-methyltransferase. Arch Biochem Biophys 645:100–106. doi:10.1016/j.abb.2018.03.019 29574051

[27] Peng M, Li C-Y, Chen X-L, Williams BT, Li K, Gao Y-N, Wang P, Wang N, Gao C, Zhang S, Schoelmerich MC, Banfield JF, Miller JB, Le Brun NE, Todd JD, Zhang Y-Z. 2022. Insights into methionine S-methylation in diverse organisms. Nat Commun 13:2947. doi:10.1038/s41467-022-30491-5 35618717 PMC9135737

[28] Li C-Y, Crack JC, Newton-Payne S, Murphy ARJ, Chen X-L, Pinchbeck BJ, Zhou S, Williams BT, Peng M, Zhang X-H, Chen Y, Le Brun NE, Todd JD, Zhang Y-Z. 2022. Mechanistic insights into the key marine dimethylsulfoniopropionate synthesis enzyme DSYB/DSYB. mLife 1:114–130. doi:10.1002/mlf2.12030 38817677 PMC10989797

[29] Trottmann F, Ishida K, Ishida-Ito M, Kries H, Groll M, Hertweck C. 2022. Pathogenic bacteria remodel central metabolic enzyme to build a cyclopropanol warhead. Nat Chem 14:884–890. doi:10.1038/s41557-022-01005-z 35906404 PMC9359912

[30] Klaus JR, Coulon PML, Koirala P, Seyedsayamdost MR, Déziel E, Chandler JR. 2020. Secondary metabolites from the Burkholderia pseudomallei complex: structure, ecology, and evolution. J Ind Microbiol Biotechnol 47:877–887. doi:10.1007/s10295-020-02317-0 33052546 PMC7746414

[31] Nwasike C, Ewert S, Jovanovic S, Haider S, Mujtaba S. 2016. SET domain-mediated lysine methylation in lower organisms regulates growth and transcription in hosts. Ann N Y Acad Sci 1376:18–28. doi:10.1111/nyas.13017 26919042

[32] Abdelraheem E, Thair B, Varela RF, Jockmann E, Popadić D, Hailes HC, Ward JM, Iribarren AM, Lewkowicz ES, Andexer JN, Hagedoorn P-L, Hanefeld U. 2022. Methyltransferases: functions and applications. Chembiochem 23:e202200212. doi:10.1002/cbic.202200212 35691829 PMC9539859

[33] Sun W, Justice I, Green EM. 2024. Defining biological and biochemical functions of noncanonical SET domain proteins. J Mol Biol 436:168318. doi:10.1016/j.jmb.2023.168318 37863247 PMC10957327

[34] Jacob Y, Bergamin E, Donoghue MTA, Mongeon V, LeBlanc C, Voigt P, Underwood CJ, Brunzelle JS, Michaels SD, Reinberg D, Couture J-F, Martienssen RA. 2014. Selective methylation of histone H3 variant H3.1 regulates heterochromatin replication. Science 343:1249–1253. doi:10.1126/science.1248357 24626927 PMC4049228

[35] Wilson JR, Jing C, Walker PA, Martin SR, Howell SA, Blackburn GM, Gamblin SJ, Xiao B. 2002. Crystal structure and functional analysis of the histone methyltransferase SET7/9. Cell 111:105–115. doi:10.1016/s0092-8674(02)00964-9 12372304

[36] Altschul SF, Gish W, Miller W, Myers EW, Lipman DJ. 1990. Basic local alignment search tool. J Mol Biol 215:403–410. doi:10.1016/S0022-2836(05)80360-2 2231712

[37] Brett PJ, DeShazer D, Woods DE. 1998. Burkholderia thailandensis sp. nov., a Burkholderia pseudomallei-like species. Int J Syst Bacteriol 48:317–320. doi:10.1099/00207713-48-1-317 9542103

[38] Dillon SC, Zhang X, Trievel RC, Cheng XD. 2005. The SET-domain protein superfamily: protein lysine methyltransferases. Genome Biol 6:227. doi:10.1186/gb-2005-6-8-227 16086857 PMC1273623

[39] Trievel RC, Beach BM, Dirk LMA, Houtz RL, Hurley JH. 2002. Structure and catalytic mechanism of a SET domain protein methyltransferase. Cell 111:91–103. doi:10.1016/s0092-8674(02)01000-0 12372303

[40] Zhang X, Yang Z, Khan SI, Horton JR, Tamaru H, Selker EU, Cheng XD. 2003. Structural basis for the product specificity of histone lysine methyltransferases. Mol Cell 12:177–185. doi:10.1016/s1097-2765(03)00224-7 12887903 PMC2713655

[41] Dunbar KL, Scharf DH, Litomska A, Hertweck C. 2017. Enzymatic carbon-sulfur bond formation in natural product biosynthesis. Chem Rev 117:5521–5577. doi:10.1021/acs.chemrev.6b00697 28418240

[42] O’Hagan D, Schmidberger JW. 2010. Enzymes that catalyse S_N_2 reaction mechanisms. Nat Prod Rep 27:900–918. doi:10.1039/b919371p 20372740

[43] Liscombe DK, Louie GV, Noel JP. 2012. Architectures, mechanisms and molecular evolution of natural product methyltransferases. Nat Prod Rep 29:1238–1250. doi:10.1039/c2np20029e 22850796

[44] Minor W, Cymborowski M, Otwinowski Z, Chruszcz M. 2006. HKL-3000: the integration of data reduction and structure solution – from diffraction images to an initial model in minutes. Acta Crystallogr D Biol Crystallogr 62:859–866. doi:10.1107/S0907444906019949 16855301

[45] Winn MD, Ballard CC, Cowtan KD, Dodson EJ, Emsley P, Evans PR, Keegan RM, Krissinel EB, Leslie AGW, McCoy A, McNicholas SJ, Murshudov GN, Pannu NS, Potterton EA, Powell HR, Read RJ, Vagin A, Wilson KS. 2011. Overview of the CCP4 suite and current developments. Acta Crystallogr D Biol Crystallogr 67:235–242. doi:10.1107/S0907444910045749 21460441 PMC3069738

[46] Jumper J, Evans R, Pritzel A, Green T, Figurnov M, Ronneberger O, Tunyasuvunakool K, Bates R, Žídek A, Potapenko A, et al.. 2021. Highly accurate protein structure prediction with AlphaFold. Nature 596:583–589. doi:10.1038/s41586-021-03819-2 34265844 PMC8371605

[47] Adams PD, Afonine PV, Bunkóczi G, Chen VB, Davis IW, Echols N, Headd JJ, Hung L-W, Kapral GJ, Grosse-Kunstleve RW, McCoy AJ, Moriarty NW, Oeffner R, Read RJ, Richardson DC, Richardson JS, Terwilliger TC, Zwart PH. 2010. PHENIX: a comprehensive Python-based system for macromolecular structure solution. Acta Crystallogr D Biol Crystallogr 66:213–221. doi:10.1107/S0907444909052925 20124702 PMC2815670

[48] Emsley P, Lohkamp B, Scott WG, Cowtan K. 2010. Features and development of Coot. Acta Crystallogr D Biol Crystallogr 66:486–501. doi:10.1107/S0907444910007493 20383002 PMC2852313

